# Three dimensional reliability analyses of currently used methods for assessment of sagittal jaw discrepancy

**DOI:** 10.4317/jced.54578

**Published:** 2018-04-01

**Authors:** Bushra-Sufyan Almaqrami, Maged-Sultan Alhammadi, BaoChang Cao

**Affiliations:** 1 Post graduate student, Orthodontics and Dentofacial Orthopedics, College of Dentistry, Lanzhou University, Lanzhou, China; 2 Assistant professor, Department of Preventive Dental Sciences, Division of Orthodontics and Dentofacial Orthopedics, College of Dentistry, Jazan University, Jazan, Saudi Arabia; 3 Professor, Department of Orthodontics and Dentofacial Orthopedics, College of Dentistry, Lanzhou University, Lanzhou, China

## Abstract

**Background:**

The objective of this study was to analyse three dimensionally the reliability and correlation of angular and linear measurements in assessment of anteroposterior skeletal discrepancy.

**Material and Methods:**

In this retrospective cross sectional study, a sample of 213 subjects were three-dimensionally analysed from cone-beam computed tomography scans. The sample was divided according to three dimensional measurement of anteroposterior relation (ANB angle) into three groups (skeletal Class I, Class II and Class III). The anterior-posterior cephalometric indicators were measured on volumetric images using Anatomage software (InVivo5.2). These measurements included three angular and seven linear measurements. Cross tabulations were performed to correlate the ANB angle with each method. Intra-class Correlation Coefficient (ICC) test was applied for the difference between the two reliability measurements. *P* value of < 0.05 was considered significant.

**Results:**

There was a statistically significant (*P*<0.05) agreement between all methods used with variability in assessment of different anteroposterior relations. The highest correlation was between ANB and DSOJ (0.913), strong correlation with AB/FH, AB/SN/, MM bisector, AB/PP, Wits appraisal (0.896, 0.890, 0.878, 0.867,and 0.858, respectively), moderate with AD/SN and Beta angle (0.787 and 0.760), and weak correlation with corrected ANB angle (0.550).

**Conclusions:**

Conjunctive usage of ANB angle with DSOJ, AB/FH, AB/SN/, MM bisector, AB/PP and Wits appraisal in 3D cephalometric analysis provide a more reliable and valid indicator of the skeletal anteroposterior relationship. Clinical relevance: Most of orthodontic literature depends on single method (ANB) with its drawbacks in assessment of skeletal discrepancy which is a cardinal factors for proper treatment planning, this study assessed three dimensionally the degree of correlation between all available methods to make clinical judgement more accurate based on more than one method of assessment.

** Key words:**Anteroposterior relationships, ANB angle, Three-dimension, CBCT.

## Introduction

An accurate anteroposterior assessment of jaw relationships is critically important in orthodontic diagnosis and treatment planning. To aid in diagnosing anteroposterior discrepancies, cephalometric analysis has incorporated various angular and linear measurements. The most commonly used skeletal sagittal discrepancy indicator so far is ANB angle, but because angular measurements are geometrically sensitive and can give false result (1-7). In addition to that, the anteroposterior relationship of the dental arch and jaw-base fail to match in at least one out of every three individuals and that linear measurement of anteroposterior jaw-base relationships is a more valid reflection of the dental arch relationship than angular measurements (8).

Many linear measurements have been introduced to overcome these shortcomings. An absolute measurement of the distance between points A (subspinale) and B (supramentale) projected onto the SN (Sella-Nasion) line was suggested by Taylor and termed as SN-AB (1). Beatty (2) used the SN line as a reference for measuring the linear perpendicular distance between A-point and D-point (cross section of symphysis).

Later on, Jacobson (6,7) used Wits appraisal (abbreviation of the University of Witwatersrand, Johannesburg. South Africa) and his method involves drawing perpendicular lines from points A and B on the maxilla and mandible respectively to the FOP (Functional Occlusal Plane). Chan (9) used the Frankfort Horizontal (FH) as a reference plane for measuring the linear perpendicular distance between A-point and B-point. Other attempts were further made to identify an appropriate reference line by Nanda and Merrill (10) using palatal plane and projections from point A and B to this plane.

Hall Scott and Ferrario (11) measured the distance between perpendiculars drawn from the bisector of the maxillomandibular plane angle to points A and B. The renewed quest for identifying anteroposterior maxillo-mandibular relationship led Al-Hammadi *et al.* (12) ,to develop dento-skeletal overjet (DSOJ); this depends on two basic concepts; the first is the dentoalveolar compensation for underlying skeletal base relation, and the second is the overjet that remains due to the incomplete dentoalveolar compensation as a result of large skeletal discrepancy.

Baik and Ververidou (13) suggested another method for determining true sagittal apical base termed as Beta angle; this angle does not depend on any cranial landmarks or dental occlusion. It is formed between the last perpendicular line from point A to C-B line (condylion to B point), and A-B line. The Conjunctive usage of different methods has been recommended for the assessment of the anteroposterior jaw discrepancy in individual patients (14-16). Measuring values that assess sagittal discrepancy has been widely studied with conventional radiographic registers in 2D but not in 3D using CBCT. This technology introduces a vision and measurement of all the craniofacial structures and cephalometric measurements accurately and completely. It allows clinicians to create new reference planes that are formed by three instead of two points. The greater accuracy of the measurements opened the door to reassessing all the measurements which were previously established (17).

The aim of this study was to use the current 3D technology with CBCT to analyze the reliability and the degree of correlation of the angular measurements like ANB, Corrected ANB, BETA angle as well as the linear measurements which include WITS appraisal, AB/PP, AB/FH, AB/SN, AD/SN, AB/MM bisecting plane angle and dento-skeletal overjet.

## Material and Methods

This is a retrospective cross sectional study approved by the ethical committee of the stomatology hospital of the University of Lanzhou, Republic of China. The sample size was calculated using a power analysis based on mean Beta angle value of Class I occlusion (31.1° ± 2°) (13) to detect a difference of 2±0.5° from this mean value with 95% power, we required a sample size of 23 in each skeletal class based on the ANB angle, and this number was doubled to a minimum 46 patients in each group.

The final sample was composed of 213 CBCTs of Chinese patients (104 males and 109 females). In spite of the fact that the sample was large, the drawback of carrying out a study of these characteristics is that irradiating patients only for research purposes is not justified, records of patients who had previously undergone a CBCT as a diagnostic tool because some additional alteration (agenesis, impaction or supernumerary teeth) were used.

All patients were selected randomly from the data base of patients at College of Dentistry, Lanzhou University, Republic of China. The inclusion criteria include (1) age above 14 years old, (2) no previous history of trauma, (3) no history of orthodontic treatment or orthognathic surgery, (4) absence of severe skeletal asymmetries or congenital defects.

Based on ANB angle, the sample was divided into 106 skeletal class I, 46 skeletal class II and 64 skeletal class III. Each of the patients had undergone a scan using the i-CAT® (Imaging Sciences International, Hatfield,PA) equipment. This CBCT device uses an amorphous sili¬con flat panel sensor to capture the fields of view (FOV). The FOV employed was the portrait mode that captures data in extended FOV mode and includes the full head of 15.0 cm (diameter) by 22 cm (height) field of view with a scanning time of 14.9 seconds. It generates a total of 544 slices with an image matrix size of 640x640. The voxel size is of 0.4 mm. The focal size is 0.5mm, and the size of its base is 119x142 cm. Tube voltage is 120 kV, and its intensity is 20.27 mAs. The size of the data files generated is in the order of 168 megabytes.

**Figure 1:**
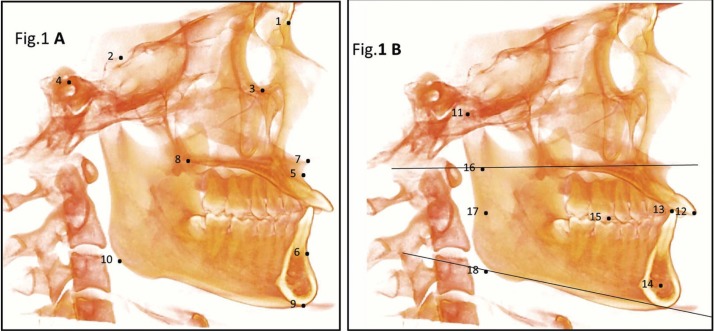
Three dimensional landmarks used in the study: (A) (1) N (nasion); (2) S (sella); (3) Or (orbitale); (4) Po (porion); (5) A (subspinale); (6) B (supramentale); (7) ANS (anterior nasal spine); (8) PNS (posterior nasal spine); (9) Me (menton); (10) Go (gonion). (B) (11) Co (condylion); (12) UIR (incisal edge of upper right central incisor); (13) LIR (incisal edge of the lower right central incisor); (14) D point; (15) U16, U26 (upper right and left 1st molar), L46, L36 (lower right and left 1st molar); (16) Bi1 point; (17) Bi2 point and (18) Bi3 point.

The raw data obtained from the CBCTs were imported to the InVivo5.2 software (Anatomage, San Jose, CA) which was used to visualize the slices and 3D images that are obtained from a CBCT. This is where the 3D reconstruction of the DICOM images (Digital Imaging and Communications in Medicine) is made. Nineteen cephalometric points were defined on each of the three spatial planes (X, Y, Z). All landmarks were initially digitized in 3D volume and finally localized by slice locator ( Table 1, Fig. 1 A,B).

**Table 1:**
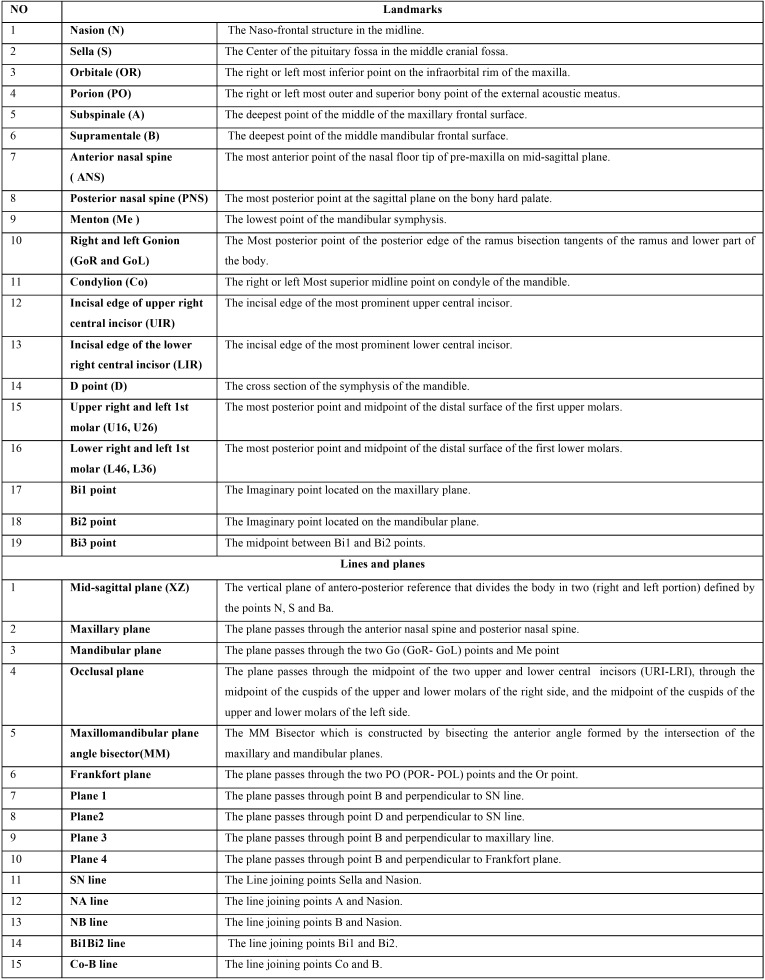
Definitions of anatomical landmarks, three-dimensional lines and planes for cone beam computed tomography analysis.

**Figure 2:**
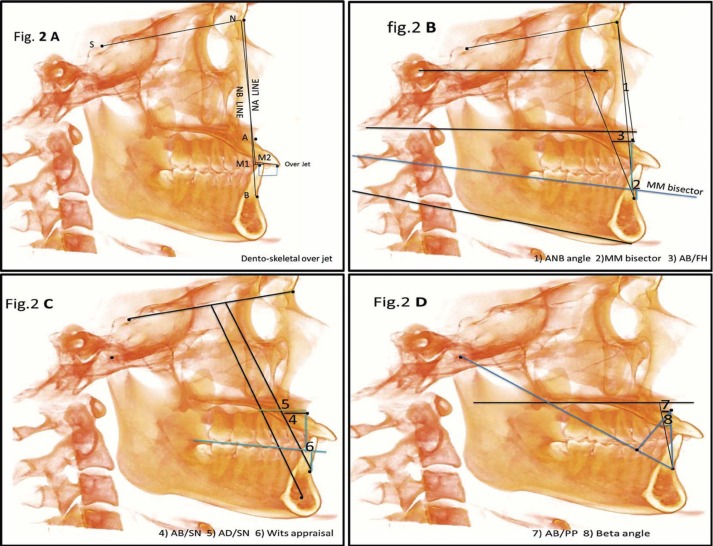
Three dimensional measurements used in the study: (A) Dento-skeletal over jet; (B) ANB angle; MM bisector AB/FH. (C) AB/SN; AD/SN; Wits appraisal. (D) AB/PP; Beta angle.

The next step was to design a 3D cephalometric analysis of the maxillo-mandibular relationship. All reference lines and planes were described in ( Table 1). All three dimensional skeletal measurements are outlined in ( Table 2, Fig. 2) this include ANB angle which classified the sagittal relation between the maxillary and mandibular skeletal bases into skeletal class I (0 ˂ ANB ˂ 4.7°) ; class II ( ANB ˃ 4.7° ) and class III (ANB<0.7°); Corrected ANB angle (ANB*); Wits Appraisal; MM bisecting plane angle; Beta Angle; Dentoskeletal Overjet ; A-Plane 1; A-Plane 2; A-Plane 3; and A-Plane 4.

**Table 2:**
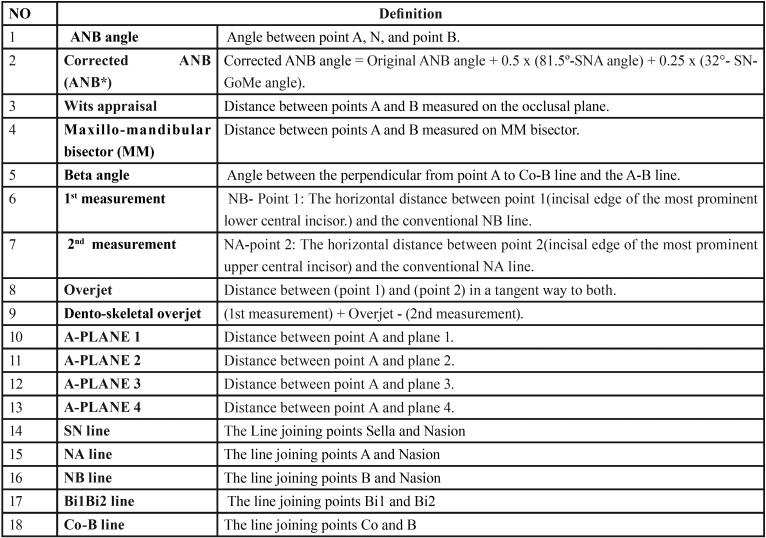
Definitions of anatomical landmarks, three-dimensional lines and planes for cone beam computed tomography analysis.

To determine the reliability of results, random selection of 10% of the total examined sample (20 CBCTs) were measured twice within a 2-week interval with the same observer and by another observer. Data entered and analyzed using the statistical package for social services (SPSS) Version 21 (Armonk, NY: IBM Corp.) for windows software. Intra-class correlation coefficient (ICC) test was applied for the difference between the two reliability measurements. Kappa statistics and cross tabulation were performed to correlate the gold standard with other measurement methods. *P* value of < 0.05 was considered significant.

## Results

The results showed a very good intra- and inter-examiner reliability (Alpha range: 0.991 – 1 and 0.993-1) respectively. Cronbach’s alpha reliability coefficient normally ranged between 0 and 1. The closer a Cronbach’s alpha coefficient is to1.0, the higher reliability. An alpha of 0.8 is probably a reasonable goal. In the present study, a statistically significant correlation was found (*p*< 0.001) between all methods ( Table 3). The highest degree of accordance between ANB angle in skeletal class 1 was with AB/FH (82.1%) while the lowest was with AB/SN (37.7%). In skeletal class II the highest degree of accordance was found between ANB angle and maxillo-mandibular bisector (97.8%) while the lowest was with corrected ANB (45.7%). Regarding skeletal class III the highest degree of accordance was existed between ANB and AB/SN (98.4%) while the lowest one was with Maxillo-mandibular bisector (45.9%).

**Table 3:**
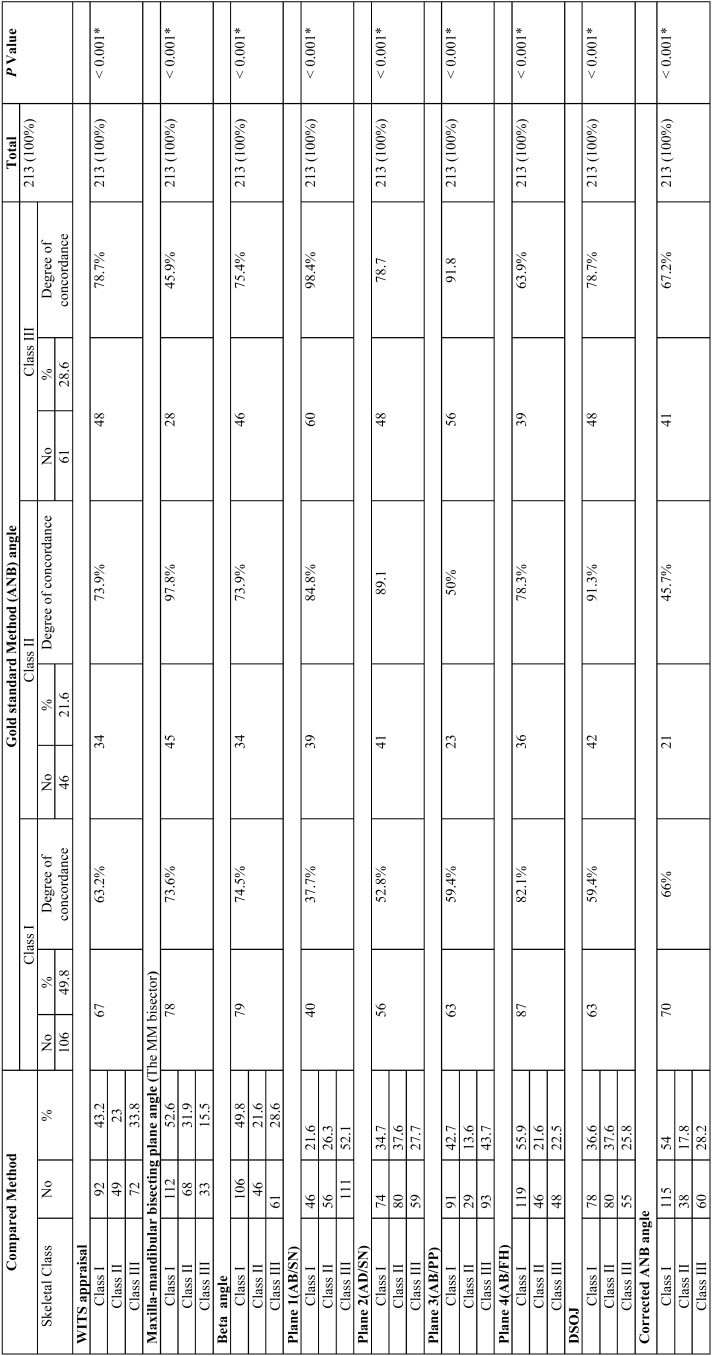
Kappa statistics and cross tabulation of all categories for tested methods relative to the gold standards.

Overall ANB angle showed a strong positive correlation with all the other variables ( Table 4). The inter-class correlation coefficient reveals that the highest correlation was between ANB and DSOJ (0.913), strong correlation with AB/FH, AB/SN/, MM bisector, AB/PP, Wits appraisal (0.896, 0.890, 0.878, 0.867, 0.858, respectively), moderate with AD/SN and Beta angle (0.787 and 0.760), and weak correlation with corrected ANB angle (0.550).

**Table 4:**
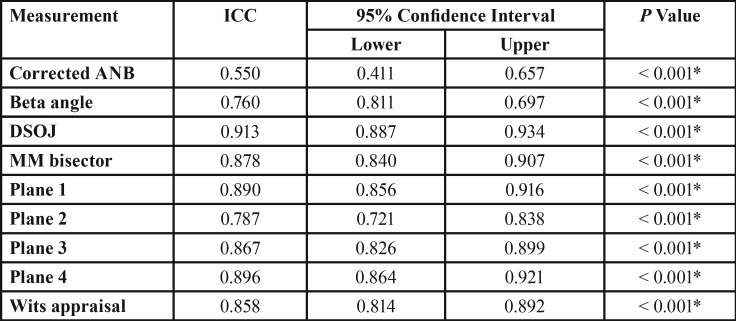
Intra-class Correlation Coefficient (ICC) of tested method relative to the gold standard.

## Discussion

Despite the fact that there are already a lot of studies on this issue, however, possibly until now there is no comprehensive study for assessment of methods analysing anteroposterior jaw relationship using 3D technology with CBCT. In this study, repeat tracings of 20 CBCTs designed to test the reliability of the tested methods showed high reliability of all measurements, and this is may be due to high accuracy of the CBCT systems in the spatial location of cephalometric points. Landmarks, in 3D, were adjusted in different slices and defined (17), increasing its reliability more.

Based on the results of this study, it can be seen how the anteroposterior relationship of our sample was classified differently in different measurements which were used, and in all kinds of malocclusion. This disagreement showed that the facial skeletal relationships were more complex than usual.

Natalia *et al.* (18), also concluded the same result when classified the sample according to the sagittal relationship, the values obtained of ANB (Class I: 53%; Class II: 37%; Class III: 10%) and Wits (Class I: 35%; Class II: 56%; Class III: 9%) did not coincide, except for the Class III group. Moreover, a high percentage of patients (n=22; 49%) which classified differently (Class I and Class II patients) by ANB and Wits, had a mesiofacial pattern with a mandibular plane angle within normal values.

Oktay (19) concluded that the ANB angle was not less reliable than any other cephalometric methods as a sagittal anteroposterior parameter. For the purposes of this study, the ANB angle was used to test the validity of all other methods. Despite its shortcomings, the ANB angle provides a reference point that is familiar to most clinicians.(20)

Jarvinen (21) reported that the correlation of ANB angle with other methods like Wits appraisal, AXD angle and A-D Distance for assessment the apical base relationship was relatively low that’s ranging from 0.61 to 0.64 with the highest correlation was exist between ANB angle and Wits appraisal while the correlation between Wits and other indicators was low 0.41-0.62. In contrast to these findings, our result was found a relatively high correlation between ANB and other methods.

The overall correlation of different variables to ANB angle was varied and ranged from 0.550 to 0.913 with the highest consistent correlations were recorded between ANB angle and dento-skeletal overjet. This parameter uses the conventional NA (Nasion-point A), NB (Nasion-point B) lines and the incisolabial line angle of the most prominent lower central incisor and the incisopalatal line angle of the most prominent upper central incisor (12). The result of Kappa statistical analysis also showed good agreement between this method and ANB angle. This was in accordance with the results found by Al-Hammadi *et al.* (12).

The AF-BF parameter used the Frankfort plane as a reference plane. This plane considered a relatively unreliable reference for cephalometric analysis because of difficulties in accurate locating the landmarks on cephalograms (22). This drawback can be overcome by using the latest technological investigation like CBCT. Our result reported that the considerable high correlation (0.896) existed between ANB and AF-BF. This finding is in agreement with the findings of Oktay (19) who found that significant positive relationship between ANB angle AF-BF (0.74).

In an attempt to evaluate the influence which changes in the relative position of nasion, A and B points upon ANB angle, Taylor (1) suggested new parameter; the linear distance to be measured between point A and B’. B’ is the perpendicular from point B to the SN plane. He found that there was 1mm of change from point A to perpendicular B` for each degree of change in ANB angle. Unfortunately, there is no other study in literature that directly correlate the relation of ANB angle with AB/SN distance except this study that showed significantly high correlation between them (0.890).

Many researchers have examined the corollary relationship between the Wits analysis and the ANB angle with mixed results. Hall-Scott (11) found a correlation of 0.95 in children and 0.83 in adults between ANB angle and MM bisector. Foley *et al.* (23), found a relatively high correlation between ANB and MM bisector values in class II cases (0.852 at T3 in control subjects, 0.631 in treated subjects). Also, the same authors (23) found a correlation of (0.666 at T3 in control subjects, 0.691 in treated subject) in class I and class III cases. All these results were in accordance with our result which is that a relatively high correlation between ANB and MM bisector (0.878).

The result of this study also showed a strong correlation between ANB angle and Wits appraisal (0.858), which was in agreement with Oktay (19) who reported strong correlation between ANB angle and Wits appraisal. Singh *et al.* (24), studied the correlation between ANB angle MM bisector. Wits appraisal and ANB. They concluded that there were strong and predictable correlations between wits appraisal and ANB angle (0.840) and between the ANB angle and MM bisector (0.865). Hiroyuki *et al.* (25), also reported the correlation between ANB angle and Wits appraisal of 0.54.

Although these studies reported predictable correlations between ANB angle and wits appraisal, several other studies reported weak correlations between them (18,22,23). Nanda and Merrill (10) compared APP-BPP with ANB angle, wits appraisal, and nasion perpendicular to determine how these measures compared in diagnosing the sagittal relation between maxilla and mandible. The ANB angle and nasion perpendicular were found to be valuable while the measure to wits appraisal was found to be biased in favour of class III relationships. In this study, there was a large positive correlation between APP-BPP with ANB angle (0.867).

Beaty (2) introduced A-D distance for evaluating horizontal jaw relationship which compensates divergence of the apical base. According to our results the correlation between A-D distance and ANB angle was found to be predictable (0.787). This was in accordance with the results found by Kapoor’s (26) who compared three methods for establishing the sagittal relationships which were Wits appraisal, Wiley analysis, and A-D distance. He found a direct significant correlation between A-D distance and ANB angle before and after treatment and mentioned that the A-D distance was found to be more reliable than Wits appraisal. A-D distance was recommended to be used in determining sagittal apical base relationship (26). Jarvinen (21) also compared two angular (ANB, AXD) and two linear measurements (A-D distance, Wits appraisal) for establishing sagittal relation, and he has come to a conclusion that the highest correlation existed between AXD angle and A-D distance (0.93) which might indicate that the effect of inter-individual variation in anterior face height on AXD angle is not significant whereas the correlation between ANB angle and other measurements were relatively low (0.61-0.64).According to Jarvinen’s results, AXD angle and A-D distance were recommended to be used in determining sagittal relationships.

The results of the present study found significant correlation between ANB angle and Beta angle. These results are in accordance with Aparna *et al.* (27), who reported that there was significant relation between ANB angle and Beta angle, and between Beta angle and Wits appraisal. The same authors evaluated the correlation between ANB and Beta angle and between ANB and wits appraisal in all malocclusion groups. They found no significant relation between ANB angle and Beta angle or between Wits appraisal and Beta angle in Class I and Class III groups. While the correlation between ANB angle and Beta angle in class II was found to be significant. This finding is in agreement to the findings of Singh *et al.* (24), who reported predictable significant correlation between ANB angle and Beta angle. 

The parameters compared in the present study are similar to Ferrario *et al.* (28), who has analyzed ANB angle, corrected ANB angle, Wits appraisal and MM bisector. They found that corrected ANB was the best among the three with correlation coefficient of (0.915). This was in accordance with the results found by Saad *et al.* (29) who found high correlations (0.797) between the two parameters used to assess the sagittal jaw discrepancy. However, in our study the correlation coefficient between ANB angle and corrected ANB was relatively low (0.550).

The diversity of results in our study is due to the effects of geometric distortion in each parameter, the interchangeability between methods can be evaluated. Jacobson (7) reported that in an individual with excellent occlusion the high ANB angle could be due to clockwise rotation of the maxilla with regard to the anterior cranial base and/or a forward position of the maxilla in relation to the nasion. Consequently, when using ANB or Wits in such cases, differences were observed between both measurements. In addition to that, he explained that the ANB angle was only reliable if the mandibular plane angle was normal, however our results are in contrast to the conclusion of Jacobson; when analysing the facial pattern (based on the mandibular plane) of the 62 patients for whom the Wits and ANB did not coincide, we observe that two third of them, 41 out of 62 (66%), had a mesiofacial pattern.

## Conclusions

1. The variability of agreement between ANB angle and other methods for determining anteroposterior relation showed that the skeletal relationships are more complex than usual.

2. There was a strong correlation between ANB angle and other tested methods with 

the highest correlation was between ANB and DSOJ, strong correlation with AB/FH, AB/SN/, MM bisector, AB/PP, Wits appraisal respectively, moderate with AD/SN and Beta angle, and weak correlation with corrected ANB angle .

3- To assess the skeletal relationship, conjunctive usage of ANB angle with other methods provide a more reliable and valid indicator of the skeletal anteroposterior relation.
